# 

**Published:** 1899-08

**Authors:** 


SUBSCRIPTION $1 00.

PUBLISHED MONTHLY.

### ATLANTA JOURNAL-RECORD OF MEDICINE.

Successor to Atlanta Medical and Surgical Journal, Established 1855, and Southern Medical Record, Established 1870.

VOL. I. ATLANTA, GA., AUGUST, 1899. No. 5.

